# Patients'perspectives and motivators to participate in clinical trials with novel therapies for rheumatoid arthritis

**Published:** 2009-04-25

**Authors:** G Udrea, B Dumitrescu, M Purcarea, I Balan, E Rezus, D Deculescu

**Affiliations:** * ‘Carol Davila’ University of Medicine, BucharestRomania; ** Internal Medicine and Rheumatology Clinic, ‘Dr. I. Cantacuzino’ Clinical Hospital, BucharestRomania; *** MedLife Clinic, Bucharest Romania; **** ‘Grigore T. Popa’ University of Medicine and Pharmacy, IasiRomania; ***** Rheumatology Department, VideleRomania

## Abstract

Background and Purposes–Successful advances in the treatment of rheumatoid arthritis rely on enrolment of patients into clinical trials with novel agents. The aim of this study was to assess the patients' perspectives and motivators to participate in clinical trials.

Methods–Consecutive patients with rheumatoid arthritis attending three rheumatology departments in Romania underwent structured questionnaire interview regarding the motivation/possible causes of acceptance or drawbacks to participate in a clinical trial.

Results–A total of 96 patients, mean age 48, 30% men 70% women answered. Response rate was 95%. Previous participation in other clinical trials was 23%. Patients were highly motivated to participate in order to help themselves or other patients and to enhance the knowledge about the disease. Patients were prone to ask for advice about their enrolment in the study from the family and their current physicians, including the general practitioner. The need for supplementary information about the study was felt because they had not dared to ask for the information, although they trusted their current doctor. A high percentage considered payment and free complete blood tests as a stimulus, especially among patients with lower levels of education (p=0.03, Fisher's ANOVA). Advertising for investigational medical product for purposes of patient recruitment was important for 57%, not only for safety or trust, but also for transparency and as a tool to get information. 73% of the persons agreed to the usefulness of patients association. 26% of them were willing to be actively involved, especially to report and include adverse events in the study settings. 58% were motivated if they knew other patients were consulted. Patients were not motivated because of the adverse events, placebo effect, treatment discontinuation, limited previous experience, availability of alternative therapies and doctor reimbursement for the study.

Conclusions–The current study suggests that awareness of factors (positive and negative) which influence motivation to participate in a clinical trial may help to refine patient's education and to consider new strategies for future trials.

## Introduction

The clinical research trials with novel therapies are intended to bring benefits to society and future patients by advancing medical knowledge. The research done has increased dramatically in the past 15 years, as researchers in the public and private sectors have strived to develop and bring to the public a wider range of diagnostic tests and treatment than ever. Thus, more patients than ever are needed to participate in trials. Although the general public expects and demands that the biomedical community should develop new, safe and effective approaches to the treatment of different diseases, the same public is not aware of the important role that public participation plays in the development of medical advances. From this point of view patient accrual to clinical trials is a difficult problem [1], [2,4]

In the mean time, although ‘new’ often implies ‘better’, the fact is that until clinical research on a new treatment is complete, we do not know if it works better, the same as, or worse than already available standard therapies. The researches cannot guarantee that the treatment under investigation will provide a benefit. Weighing the risk and benefits to make an informed decision about whether to participate in a clinical trial or not can often be complicated. Altruism is cited by many to be the major motivating factor for participating in clinical research [5] but may not be the sole motivating factor [6]. A lot of studies have investigated the motivations and inhibiting factors for patients participating in phase 1 and phase 2 cancer clinical trials [7], but there are only a few assessing the patients with rheumatoid arthritis and their perspectives regarding the participation in the research.

Our study aims to assess the patients' perspectives and motivators to participate in clinical trials.

## Material and methods

Consecutive patients with rheumatoid arthritis attending three rheumatology departments during the study period (October 2007–March 2008) were invited to take part in this questionnaire survey. The local ethical committee approved the study design and the final format of the questionnaire.

The survey's investigators asked the patients if they agreed to participate in a questionnaire survey that would take about 15 minutes to be completed. Each patient underwent a structured interview using a questionnaire designed to assess the patients' perspectives and motivators to participate in clinical trials with novel biologic agents. We stressed that participation was voluntary and that all information would be treated in confidence. The questionnaire was completed in the absence of the research nurse and was delivered to the clinic by each individual.

## Study instruments

The questionnaire was developed by authors and was piloted with 10 other different patients to ensure the clarity of meaning. The final format of the questionnaire consisted of 13 questions (open and close–ended questions). The structured questionnaire is available on request. Parameters such as sex, age, marital status (single, married, separated/divorced/widowed), employment (full/part–time employment, unemployed, housewife, retired), educational level (high school, college, university/postgraduate), disease duration, were also recorded.

## Statistical analysis

The primary statistical analysis was intended to be descriptive. Continuous variables were described as the mean plus/minus SD (standard deviation). Categorical variables were reported as percentages. The chi-square test was used to analyze categorical variables. One way ANOVA was used to investigate differences between means. P<0.05 was considered statistically significant. Data was analyzed using SPSS for Windows (version 13.0 Program).

## Results Participants

96 consecutive and consenting patients with rheumatoid arthritis agreed to participate in the survey (70% women, 30% men). Mean age was 48 plus/minus 13 years old, and mean disease duration was 11 plus/minus 9 years. 37% of them were retired. Most of them had medium educational level (73%). The response rate was 95% (96 out of 101 patients). Table 1 shows the demographic characteristics of the participants.

**Table 1:** Demographics of the patients (N=96)

Characteristics	Number	Percent
Gender
Male	29	30
Female	67	70
Age group (years old)
20–29	13	13
30–39	13	13
40–49	16	16
50–59	35	36
60–69	19	20
70–79	0	0
Marital status
Single	11	11
Married	80	83
Separated/divorced/widowed	5	5
Education
High school	58	60
College	12	13
University/Postgraduate	26	27
Employment
Full/part–time employment	41	43
Unemployed	3	3
Housewife	16	17
Retired	36	37
Previous trial participation
Yes	22	23
No	74	77

## Previous participation in clinical trials

23% of the patients were involved in previous randomized controlled clinical trials with medication for rheumatoid arthritis. All of them have signed a written informed consent. Only 87% of them received a copy of the written informed consent for the general practitioner. When they had been asked about ‘How much time did you have to read it?’ they answered: 14% (13) less than 10 minutes, 29% (28) less than 1 hour, 57% (55%) one day. 47% of them felt the need for advice. 37% of them asked the general practitioner for advice, 30% asked the family, 23% other patients and 17% pharmacists. They had been asked ‘Why did you ask for advice?’ 10% of them asked for advice because they needed more information about the trial, 13% mentioned fear of ‘unknown’ and 17% of them reported the need for reassurance in making the decision. Other reasons were mentioned as well: ‘I need a second opinion from someone not involved’, ‘I didn't understand the medical language that doctor used’, I thought of being ‘ guinea pigs’. Previous clinical trials participation was greater in patients retired (p=0.04).There was no significant correlation between disease duration (p=0.2) and educational level (p=0.5). These patients were motivated by the desire to help gather data about the disease (p=0.013) and they were also prone to be involved in establishing objectives in future clinical trials. (p=0.04).

## Motivators for future participation in clinical trials

All participants answered the closed question ‘Can you tell us the reasons why you would participate in a future clinical trial?’. Most of them (90%) indicated hope of the health benefit as the most important motivating factor in their decision to participate in the trial. Many participants gave altruistic reasons as ‘helping others to get better’(77%) or ‘helping in gathering new data about the disease’ (83%). Other important motivating reasons were the ‘trust in the physician’ (72%) and ‘easy access to free complete laboratory tests’(63%). Only a few patients pointed on ‘getting paid for participation’ (27%), and ‘maintaining a good relation with the doctor’ (17%) as possible reasons to participate in a clinical trial. Patients with lower levels of education evoked free blood tests(63%) and payment(27%) (ANOVA, p=0.03, p=0.0004) as motivators for future participation in clinical trials.

**Table 2:** Motivator for participation in clinical trials

Question	Yes	Percent
Can you tell us the reasons why you would participate in a future clinical trial?
help me to get better	86	(90)
help others to get better	74	(77)
gather new data about the disease	80	(83)
getting paid for participation	26	(27)
easy access to free complete laboratory tests	60	(63)
maintain a good relation with the doctor	16	(17)
trust in the physician	69	(70)

## Sources of information as motivator for clinical trials

57% of them considered that media promotion (in a newspaper, on the internet, on TV) of the clinical trial would raise clinical trial participation because it would make them more confident about it (37%).

73% of them considered that being aware and receiving information about clinical trials from social leagues would improve the patients ' participation in clinical trials. 58% of them mentioned that they would be more confident if they knew the patients have been consulted before the trial began. 26% of them wished they were involved in the establishment of the trial objectives and 10% in making clinical decisions regarding the trial. 17% were glad to have brought a relative to the consultation at the moment they received the information.

## Disincentives (drawbacks) for future participation in clinical trials (Table 3)

When the participants had been asked ‘Which of the following reasons would you be negatively influenced by to participate in a future clinical trial?’ the answers were: ‘potential side effects of the investigational product’ (73%), ‘the chance of being randomized to placebo’ (50%), and ‘limited previous experience with the investigational product’ (53%) These were the main reasons for being skeptic regarding the participation in the clinical trial. The ‘availability of reasonable alternative therapies’ (43%) as well as ‘treatment discontinuation at the end of the trial’ (33%) were also disincentives for participation. Only a small number of participants mentioned ‘payments to clinicians for patients' recruitment’ (13%) and ‘disruption of daily routine’(13%) as discouragements.

**Table 3:** Disincentives for participation in clinical trials

Question	Yes	Percent
Which of the following reasons would you be negatively influenced by to participate in a future clinical trial?
Availability of reasonable alternatives therapies	45	(43)
Limited previous experience with the investigational product	55	(53)
Potential side effects of the investigational product	76	(73)
The chance of being randomized to placebo	52	(50)
Treatment discontinuation at the end of the trial	34	(33)
Too much blood drawn during the trial	14	(13)
Disruption of daily routine	14	(13)
Transportation problems	14	(13)
Payments to clinicians for patients recruitment	14	(13)

## Discussion

Attitude towards clinical research was positive. Both personal and altruistic motives for participation were highly rated. Just like in other studies [8], the patients stated their motivation to participate was to help others, to improve their own health, and to contribute to medical science but also the trust in the doctor's request. Our data also showed the need for education programs in order to raise awareness, reduce fears, and dispel myths about clinical trials [9]. Another aspect that arose is the importance of the advertisement of research studies that may increase patient participation rates [10]. In the same time, giving people more information and more time to reflect tends to be associated with a lower consent rate. Although divulging less information seems to be associated with less anxiety, there is evidence of an interaction with knowledge–high levels of knowledge are significantly associated with less anxiety. There is some evidence to suggest that there is an optimal amount of information which enhances patient's understanding and which, in turn, reduces anxiety [11].

The most common reasons given for unwillingness to participate were the ones concerning the trial setting; a dislike of randomization, presence of a placebo group, potential side effects. The fear of the unknown and resentments towards randomization as primary reasons for nonparticipation were also documented [12]. A number of patients had a negative response to the placebo. Previous research has documented negative attitude about placebo, as a reason for a low rate of enrolment in clinical trials [13], [ 14], [15]. This fact could be reasonably managed by showing that patients on placebo will receive the standard care for their stage of disease. A minority was concerned with potential conflicts of interests. The clinical research funding mechanisms and the business of clinical research are also aspects which should be discussed with the participants in clinical trials, in order to build trust with the researchers and help participants to feel more comfortable and confident to participate in the research [ 16].

A small number of patients mentioned paying to participate in clinical trials as a motivator factor, but this aspect is ethically controversial. Halpern et co. showed there is a positive correlation between income and the influence of the willingness to participate in a clinical trial, but this is true especially among the wealthier people [17]. Our results show that access to free laboratory investigation is motivating. This prompts to some possibly health system issues that may vary from country to country. In existing UK guidelines, the issues around payments to clinicians or patients are implied rather than stated, usually linked to a discussion or a conflict of interest and disclosure of any such conflicts. Interviews with NHS health professionals, mainly research active clinicians, indicated concerns over the likely effects of payment. While reimbursement of expenses incurred to do research was strongly supported, payment to stimulate recruitment was not. Direct payment to clinicians, linked to recruitment or to research involvement was rare in publicly funded trials. A code of practice for any such payments was suggested, closely linked to the principles of Good Clinical Practice in research. Other factors such as interest in the topic, scope for patient benefit and good communication were considered the most important motivations for research involvement. Interviews with the public indicated low levels of awareness of payments to clinicians linked to patient involvement in trials, and unanimous support for full disclosure of any such payments. Interviews with research managers in the pharmaceutical industry showed greater familiarity with payments for research involvement, which, in recent years, has shifted to payment to institutions rather than individual clinicians [18].

Study drawbacks include the fact that patients were drawn from three different particular departments of rheumatology and there was a limited number. Further research is undoubtedly useful in determining whether our findings can be generalized to other rheumatic diseases or to populations with other cultural beliefs about health care, research, and disability.

The current study suggests that awareness of factors (positive and negative) which influence motivation to participate in a clinical trial may help refine patient education and consider new strategies for future trials. Patients' enrolment in clinical trials may be increased by heightened physician awareness of such predictors and enhanced communication between physician and patients. Further research is needed to evaluate strategies to better inform patients about clinical trials. As our study outlined the Social Leagues of the patients with rheumatic diseases have an important role in the improvement of the enrolment of the patients with rheumatic diseases in the research. There are also studies that outline the importance of a strong partnership developed between researchers (academic institutions) and communities of patients with specific diseases. This partnership could provide an infrastructure that supports the interest of both groups [19].
